# Supplementary material for: The causal role of circulating inflammatory markers in osteoporosis: a bidirectional Mendelian randomized study
Source: Front Immunol. 2024 Jul 18;15:1412298. doi: 10.3389/fimmu.2024.1412298 (PMC11291241; doi:10.3389/fimmu.2024.1412298)
Supplement: Supplementary file 1 [file DataSheet_1.zip › supplementary material/CIM-OSTEOPOROSIS /GCST90274845.csv_leave_one_out.pdf]

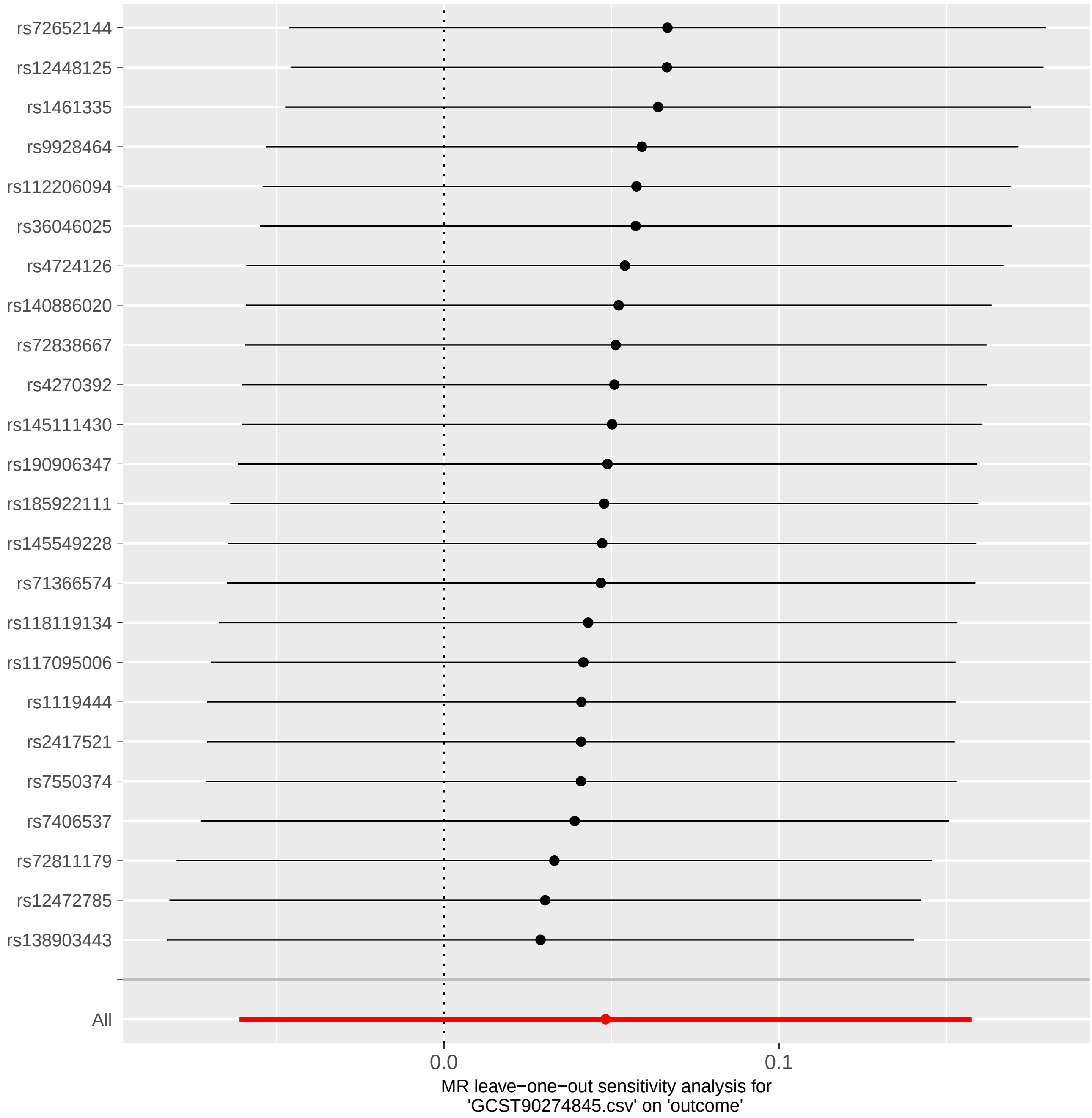

rs72652144
rs12448125
rs1461335
rs9928464
rs112206094
rs36046025
rs4724126
rs140886020
rs72838667
rs4270392
rs145111430
rs190906347
rs185922111
rs145549228
rs71366574
rs118119134
rs117095006
rs1119444
rs2417521
rs7550374
rs7406537
rs72811179
rs12472785
rs138903443
All
0.0
0.1
MR leave−one−out sensitivity analysis for
'GCST90274845.csv' on 'outcome'
